# Long-term outcomes and survival predictors in patients with ectopic ACTH syndrome: data from a retrospective cohort study

**DOI:** 10.1530/EC-25-0411

**Published:** 2025-09-22

**Authors:** Zhanna Belaya, Liudmila Rozhinskaya, Olga Golounina, Alexander Solodovnikov, Evgeniya Marova, Svetlana Arapova, Michail Pikunov, Alla Markovich, Elizaveta Mamedova, Elena Przhialkovskaya, Ekaterina Pigarova, Valentin Fadeev, Nikolay Kuznetsov, Ivan Sitkin, Larisa Dzeranova, Alexander Lutsenko, Natalia Mokrysheva, Ivan Dedov, Galina Melnichenko

**Affiliations:** ^1^ Endocrinology Research Centre, Moscow, Russia; ^2^ Peoples’ Friendship University of Russia Named After Patrice Lumumba, Moscow, Russia; ^3^ National Medical Research Center of Surgery Named After A. V. Vishnevsky, Moscow, Russia; ^4^ National Medical Research Center of Oncology Named After N. N. Blokhin, Moscow, Russia; ^5^ I.M. Sechenov First Moscow State Medical University (Sechenov University), Moscow, Russia

**Keywords:** ectopic ACTH syndrome, neuroendocrine tumor, hypercortisolism, survival analyses, mortality

## Abstract

**Background:**

Ectopic ACTH syndrome (EAS) is caused by non-pituitary neuroendocrine tumor (NET) that produces adrenocorticotropic hormone (ACTH).

**Objective:**

To identify survival predictors and to analyze long-term outcomes in patients with EAS.

**Methods:**

Medical records of patients with verified EAS between 1990 and 2024 were analyzed to obtain the initial clinical and biochemical data along with subsequent interventions and survival outcomes.

**Results:**

The study included 173 patients (107 women and 66 men), with a median (Q25–Q75) age of 42 years (29; 55). The median follow-up period was 54 months (16; 99) with a maximum of 402 months. Over the observation period, death was registered in 50 (28.9%) cases. The overall 3- and 5-year survival rates were 77 and 70%, respectively. Multivariable analysis revealed the following negative predictive factors for survival: age at diagnosis ≥51 years (hazard ratio (HR) 3.53; 95% confidence interval (CI): 1.67–7.5; *P* = 0.001), presence of metastases (HR 2.93; 95% CI: 1.35–6.32; *P* = 0.006), and active hypercortisolism (HR 5.58; 95% CI 1.62–19.24; *P* = 0.006) along with late night salivary cortisol levels (LNSC) above 130 nmol/L (HR 2.81; 95% CI: 1.30–6.07; *P* = 0.009).

**Conclusion:**

Active hypercortisolism, high LNSC, distant metastases and older age at diagnosis are factors associated with mortality in EAS. As severity of hypercortisolism is the main targetable factors, it should be the focus of intervention and further studies aimed at improving outcomes.

## Introduction

Ectopic ACTH syndrome (EAS) is a paraneoplastic syndrome caused by non-pituitary neuroendocrine tumors (NETs) that produce adrenocorticotropic hormone (ACTH). EAS represents 10–20% of ACTH-dependent Cushing’s syndrome (CS) with an incidence of 0.2–1.0 per million (1, 2, 3). Presumably the first description of EAS was recorded in 1928, when a small cell lung carcinoma was reported in postmortem examination of a woman with all symptoms of hypercortisolism (4). Since then, there have been several case series of EAS, confirming that lung NETs are the most common cause of ectopic ACTH production and less frequent causes include thymic and pancreatic NETs, pheochromocytoma, paraganglioma, medullary thyroid cancer, gut NETs and other types of extremely rare tumors (3, 5, 6, 7, 8, 9, 10, 11).

In a large cohort of gastroenteropancreatic and thoracic NETs (*n* = 918), EAS was found in 29 patients, showing a prevalence of 3.2% (12). Patients with EAS had worse outcomes compared with other NETs, most likely due to hypercortisolism. Chronic hypercortisolism is associated with multiple complications, including hypokalemia, hypertension, diabetes mellitus, fragility fractures, infection and major cardiovascular events, which consequently lead to increased mortality (13, 14). Endogenous hypercortisolism caused by EAS usually presents with higher cortisol levels and has more severe manifestations compared with Cushing’s disease (13, 15). Owing to the rarity of EAS, only limited data on survival predictors is present in scientific literature (3, 16).

The purpose of our study is to identify survival predictors and to analyze long-term outcomes in a Russian cohort of patients with EAS.

## Materials and methods

We obtained medical records between 1990 and May 2024 and made contact with patients to analyze outcomes. All patients were initially referred from different regions of Russia due to suspected EAS. First diagnostic evaluations were performed at local clinics to establish ACTH-dependent hypercortisolism and to differentiate CD and EAS using various tests, including pituitary MRI and 8 mg dexamethasone suppression test. Diagnostic evaluation was repeated in our hospital, which is the leading referral center in Russia for bilateral inferior petrosal sinus sampling (BIPSS).

The diagnosis of ACTH-dependent endogenous hypercortisolism was based on the following laboratory tests: increased 24 h urinary free cortisol (24 h UFC) levels and late night salivary cortisol (LNSC) and/or increased serum cortisol levels at 23:00 and/or unsuppressed serum cortisol after 1 mg overnight dexamethasone suppression test (cut-off point 50 nmol/L) along with non-suppressed morning ACTH levels. Plasma ACTH (reference range: morning 7.2–63.3 pg/mL, late-night 2–25.5 pg/mL), late-night serum cortisol (64–327 nmol/L) and LNSC (0.5–9.6 nmol/L) levels (17) were measured using ECLIA Cobas 601, 24 h UFC (100–379 nmol/L) was measured using VITROS ECi. Active hypercortisolism was diagnosed if cortisol values in the above-mentioned tests were above the reference range by at least 30%.

BIPSS with a stimulating agent (desmopressin acetate 8 μg) was used to differentiate within ACTH-dependent hypercortisolism. EAS was established when the central-to-peripheral ACTH ratio was below 2 before stimulation and below 3 after stimulation (18, 19, 20, 21, 22). The catheter position was controlled by prolactin measurements from both the petrosal sinuses and the peripheral vein. BIPSS was considered successful when the central-to-peripheral prolactin ratio was above 2 (18, 23). If the prolactin ratio was below 2 along with the ACTH ratio indicating EAS, the procedure was repeated or, if it was anatomically impossible, the patient was referred to diagnostic neurosurgery. Only patients with verified EAS were included in the study.

In all cases of reported occult NET, EAS was established based on BIPSS with prolactin central-to-peripheral ratio above 2 along with ACTH central-to-peripheral ratio below 2 before and after stimulation. In some patients, metastasis was diagnosed without primary tumor localization; these cases were also classified as occult tumor.

Various imaging techniques, such as cervical–thoracic–abdominal and pelvic spiral thin-slice computed tomography (CT), Octreoscan with ^111^In-octreotide or ^99m^Tc-tectrotide and/or positron emission tomography/computed tomography (PET/CT), using ^68^Ga-DOTA labeled somatostatin receptor ligands were performed in order to locate the ACTH-producing tumor (24, 25, 26). We repeatedly used various imaging modalities in all patients but were not able to establish tumor localization in cases of occult tumors.

Histological analyses were obtained from medical records. NET proliferative activity was determined by Ki-67 index and mitotic active areas in TEN high power fields. ACTH production was confirmed immunohistochemically as ACTH expression in the tumor cells.

The causes of death were obtained from death certificates.

### Statistical analysis

Continuous variables are reported as the mean ± standard deviation or median with interquartile range (IQR), including minimum and maximum values. Categorical variables are shown as percentages. Fischer’s exact test was employed for comparisons between categorical variables, whereas continuous variables were compared using the Mann–Whitney test. *χ*^2^ test was used to assess differences between categorical variables. ROC analysis was used to retrieve prognostic threshold values of individual predictors. The provided thresholds were chosen based on the maximum sum of specificity and sensitivity.

For univariate analysis, the log rank test identified statistically significant differences between strata (deceased vs alive), while univariate Cox proportional hazard regression calculated hazard ratios (HRs) and constructed 95% confidence intervals (CIs).

In multivariable analysis, variables with a *P*-value below 0.05 from the univariate analysis were included in the Cox proportional hazard regression model, followed by a stepwise exclusion approach based on the maximum likelihood method. Only factors with *P* < 0.05 remained in the final model.

Statistical analyses were conducted using R version 4.3.3 (https://www.r-project.org/). Packages survminer (version 0.4.9) and survival (version 3.7-0) were used for survival analysis, while package pROC (version 1.18.5) was used for quantitative data analysis through receiver–operator characteristic curves and for the determination of cut-off values. Figures were produced using the ggplot2 package (version 3.5.1).

### Ethics statement

The study was approved by the Local Ethics Committee of the Endocrinology Research Centre (approval number 12). Study was performed in accordance with the ethical standards of 1964 Declaration of Helsinki and its later amendments. All subjects signed written informed consent for depersonalized medical data usage for research purposes. For patients diagnosed in childhood, written informed consent was obtained from the participants’ parent/legal guardian and/or from them as all patients diagnosed at 17 were enrolled into study in adulthood.

## Results

### Patient characteristics

One hundred and seventy-three patients (107 women and 66 men) were included in the study. The median follow-up period was 54 months (16; 99.5) with a maximum follow-up period of 402 months. Age at the time of diagnosis ranged from 12 to 76 years (median 42 years (29; 55)). Endogenous hypercortisolism diagnosis was based on the elevated 24 h UFC levels: median (Me) – 3,011 nmol/24 h (1,778–6,700) with maximum 15,820 nmol/24 h; LNSC – 87.2 nmol/L (45.5–175.6) with maximum 687 nmol/L; morning ACTH – 143 pg/mL (104–206); and late-night ACTH – 119.8 pg/mL (91–179). Fifteen patients (8.7%) had cyclic CS.

The general characteristics of patients with various localizations of ACTH-producing NETs are summarized in Table 1. Five patients were diagnosed in childhood: thymic NET in a 12-year-old boy, lung NET in a 16-year-old girl, and one thymic and one lung NET in two 17-year-old girls. In one case of pancreatic NET in a 17-year-old girl, we diagnosed von Hippel–Lindau syndrome, c.506T>C (p.Leu169Pro). At the time of evaluation, metastatic pancreatic NET was the only clinical presentation of von Hippel–Lindau syndrome in this girl. All children survived and reached adulthood by the time of publication. We found no differences in the tumor localizations or severity of hypercortisolism between young (18–35 (*n* = 57)) and older (36+ (*n* = 116)) patients. Older patients had higher BMI and were more likely to have diabetes mellitus, arterial hypertension and cardiovascular disease than younger patients (Supplementary Table S1 (see the section on Supplementary materials given at the end of the article)).

**Table 1:** General characteristics of patients with ACTH-producing tumors of various localizations.

Tumor localization	Total number of cases	Mean age at diagnosis, years	Follow-up period, months	Surgery: number of curative cases/debulking	Distant metastases	Number of deaths (% from total cases)
	Gender (male/female)		Me [Q1; Q3] (min; max)			
Bronchial NET	107	42 ± 15	Me 61 [20; 114] (1; 402)	76/14	18	22 (20.5%)*
	46/61					
Thymic NET	18	31 ± 12	Me 64.5 [33; 77] (2; 144)	2/14	13	11 (61%)
	7/11					
Pheochromocytoma	6	47 ± 11	(24; 144)	6/0	0	0 (0%)
	1/5					
Pancreatic NET	10	42 ± 17	Me 17.5 [5.3; 57.5] (2; 162)	2/1	6	5 (50%)
	0/10					
Cecum NET	1	53	16	0/1	1	1 (100%)
	0/1					
Appendix NET	1	20	165	1/0	1	0 (0%)
	0/1					
Medullary thyroid carcinoma	1	40	12	0/1	1	1 (100%)
	0/1					
Renal NET	3	57, 65 and 65	5, 27, 69	3/0	0	0 (0%)
	0/3					
Occult tumor	26	50 ± 17	Me 38 [11; 57] (1; 134)	-	4	10 (38%)
	12/14					

NET, neuroendocrine tumor.

* 5 Cases unknown.

The most common complications of active hypercortisolism were arterial hypertension (*n* = 147, 85%), diabetes mellitus (*n* = 98, 56.6%), osteoporosis (*n* = 99, 57.2%) with low-energy fractures in 73 cases (42.2%), cardiovascular diseases (*n* = 93, 53.8%), which included steroid cardiomyopathy, arrhythmias and conduction disorders, chronic heart failure, myocardial infarction and stroke.

### Surgical treatment of neuroendocrine tumors and adrenalectomy

The primary NET was removed in 121 patients (69.9%). Surgical treatment led to remission of hypercortisolism in 74.4% of cases. Among the most common lung NETs, typical carcinoid was diagnosed in 69 cases (Ki-67 1.6 (1.0–2.8), atypical carcinoid in 20 cases (Ki-67 9.8 (6.2–11.0) and diffuse idiopathic pulmonary neuroendocrine cell hyperplasia (DIPNECH) in one case. No other lung cancers were recorded.

Bilateral adrenalectomy was performed in 47 patients (27.2%): 32 patients underwent the procedure due to life-threatening hypercortisolism, before primary tumor was localized, and in 15 cases, bilateral adrenalectomy was performed due to inoperable disease recurrence.

Regional and distant metastases were found in 44 patients (25.4%).

### Medical treatment of EAS

Seventy-five patients (43.4%) required medications to control CS and NET. Forty-three patients were treated with long-acting somatostatin analogs (SSAs): octreotide 20–80 mg (*n* = 33) or lanreotide 120 mg (*n* = 13) once every 21–28 days. The average duration of SSA treatment was 34 months (Me 27.5 (13.8; 41)). Dose escalation was required in 39.4% of cases with 9 months being the mean time until first dose escalation.

In 28 cases, inhibitors of steroidogenesis were prescribed: ketoconazole 400–800 mg/day in 27 cases and osilodrostat 10 mg/day in one case. One patient received mifepristone 300 mg/day. Twelve patients received combined treatment of both somatostatin analogs and inhibitors of steroidogenesis. Six patients received everolimus. Sixteen patients required chemotherapy (EP, Tem/Cap, mGEMOX, aranosa + bevacizumab, and PRPT in one case). Eight patients underwent non-specific radiation therapy and in one case Lutetium-peptide receptor radionuclide therapy was performed.

### Survival predictors and outcomes

Of the 173 enrolled patients, 50 (28.9%) deaths were confirmed. Five patients were lost to follow-up.

Thirty-one patients died with active hypercortisolism and three died after surgery due to complications. The primary cause of death was multiple organ failure (*n* = 27, 54%), as stated in death certificates. Clinically, in these cases, death was predisposed by progressive deterioration complicated by infection in most cases with subsequent multiple organ failure and finally cardiac arrest. Others causes included pulmonary embolism (*n* = 6, 12%), cardiovascular events (*n* = 3, 6%), COVID-19 complications (*n* = 3, 6%), acute cerebrovascular events (*n* = 2), massive postoperative bleeding (*n* = 2), disseminated intravascular coagulation syndrome (*n* = 1), and hip fracture at the age of 81 (*n* = 1), and in five cases, the cause of death is unknown. In ten out of 50 deceased patients, the primary tumor remained occult even after autopsy.

The mean time between diagnosis and death was 33 months (Me −16 months (4.8; 55)). The 3- and 5-year overall survival rates were 77 and 70%, respectively.

A comparison of demographic and clinical data between survivors and deceased patients with EAS is presented in Table 2.

**Table 2:** Characteristics of surviving and deceased patients with ectopic ACTH syndrome.

		Survived	Died	*P*-value
Parameter		*n* = 118	*n* = 50	
Male/female		47/71	15/35	0.294
Age at the time of diagnosis, years		39 (27; 53)	52 (36; 60)	0.003
Body mass index		28 (24; 32)	27 (24; 31)	0.920
Ki-67 index, %		3.0 (1.5; 7.2)	10.0 (2.9; 19)	0.008
Metastases	Yes	14 (11.9%)	30 (60%)	<0.001
	No	104 (88.1%)	20 (40%)	
**Complications in the active hypercortisolism**				
Arterial hypertension	Yes	99 (83.9%)	43 (86.0%)	0.819
	No	19 (16.1%)	7 (14.0%)	
Cardiovascular disease	Yes	55 (46.6%)	36 (72.0%)	0.004
	No	63 (53.4%)	14 (28.0%)	
Diabetes mellitus	Yes	63 (53.4%)	32 (64.0%)	0.236
	No	55 (46.6%)	18 (36.0%)	
Osteoporosis	No osteoporosis	51 (43.2%)	20 (40.0%)	0.668
	Osteoporosis without low-energy fractures	52 (44.1%)	21 (42.0%)	
	Osteoporosis with low-energy fractures	15 (12.7%)	9 (18.0%)	
**Laboratory examinations at the time of diagnosis**				
Morning ACTH, pg/mL		133 (100; 186)	195 (137; 292)	<0.001
Late-night ACTH, pg/mL		113 (79; 161)	148 (110; 258)	0.001
Late-night salivary cortisol, nmol/L		74 (42; 127)	185 (76; 456)	<0.001
24 h urinary free cortisol, nmol/24 h		2,778 (1,732; 5,501)	4,639 (2,640; 7,723)	0.004
Late-night serum cortisol, nmol/L		1,106 (833; 1,390)	1,243 (1,062; 1,587)	0.012

NET, neuroendocrine tumor; ACTH, adrenocorticotropic hormone; data presented as the median and interquartile range (Q25–Q75).

In a univariate analysis, the negative predictors of survival were aged above 51 at diagnosis, cardiovascular disease, presence of metastasis, active hypercortisolism and high LNSC and/or late-night serum cortisol and/or 24 h urinary free cortisol, and high ACTH levels.

Positive predictors of survival in the univariate regression model were bronchial NET localization and successful surgical removal of primary tumor. The overall 3- and 5-year survival rates for bronchial NETs were 85 and 78%, respectively, and for thymic NETs – 71 and 57%, respectively. Patients with occult tumor had significantly worse overall 3- and 5-year survival rates − 58%.

There were no statistically significant differences in other potential prognostic factors, such as gender, bilateral adrenalectomy, cyclic course of EAS or any typical complications of hypercortisolism – arterial hypertension, diabetes mellitus or osteoporosis with fragility fractures. The results of univariate analysis of survival predictors are presented in Table 3.

**Table 3:** Univariable analysis of survival predictors in patients with ectopic ACTH syndrome.

Variable	HR (95% CI)	*P*-value
Gender (male)	0.653 (0.356–1.196)	0.167
Age at time of diagnosis (≥51 years)	2.749 (1.570–4.816)	<0.001
NET localization (bronchial NET vs all others)	0.442 (0.234–0.838)	0.012
Presence of metastasis	4.295 (2.448–7.538)	<0.001
Cyclic course of disease	0.386 (0.094–1.589)	0.187
Successful surgical removal of primary tumor	0.204 (0.115–0.362)	<0.001
Bilateral adrenalectomy	1.148 (0.638–2.064)	0.645
Active hypercortisolism	7.070 (3.302–15.139)	<0.001
Presence of arterial hypertension	1.239 (0.557–2.759)	0.599
Presence of cardiovascular disease	2.525 (1.362–4.684)	0.003
Presence of diabetes mellitus	1.715 (0.960–3.063)	0.068
Presence of osteoporosis with fractures	1.195 (0.543–2.627)	0.658
24 hour urinary free cortisol (≥4,564 nmol/24 h or 14-fold increase above reference range)	2.283 (1.277–4.082)	0.005
Late-night serum cortisol (≥1,150 nmol/L or 3.5-fold increase above the reference range)	1.889 (1.045–3.416)	0.035
Late-night salivary cortisol (≥130 nmol/L or 14 times above the upper limit of reference range)	5.440 (2.595–11.404)	<0.001
Morning plasma ACTH (≥145 pg/mL)	3.292 (1.741–6.226)	<0.001
Late-night plasma ACTH (≥128 pg/mL)	2.208 (1.217–4.005)	0.009

NET, neuroendocrine tumor; ACTH, adrenocorticotropic hormone, HR, hazard ratio; CI, confidence interval.

**Figure 1:**
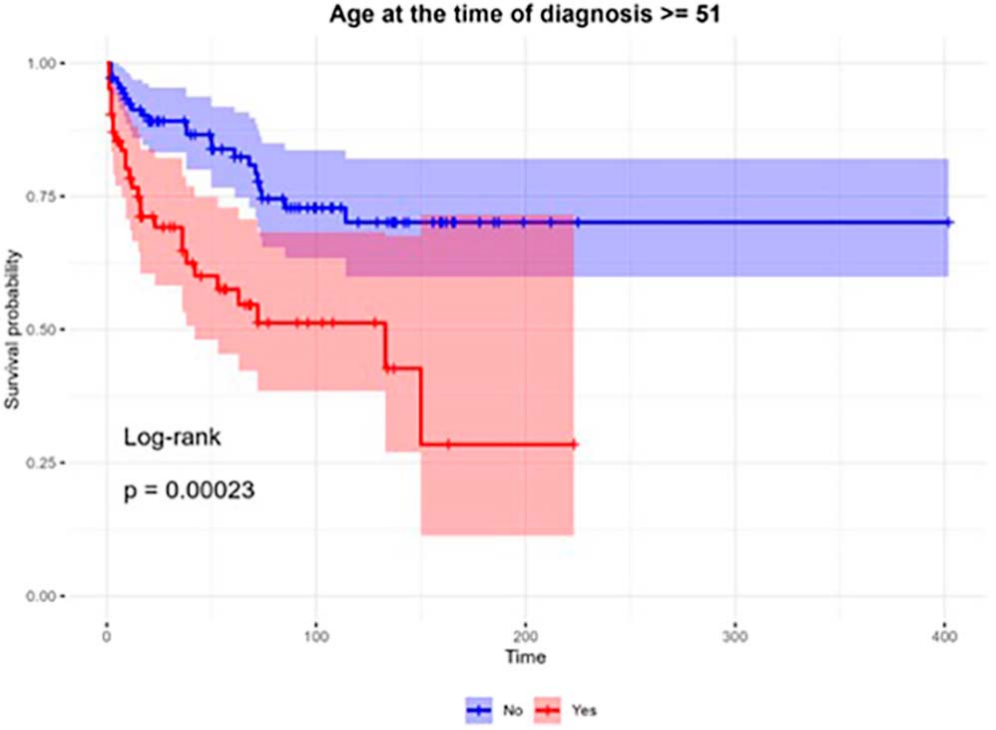
Overall survival by patient age upon diagnosis. Survival probabilities were estimated employing the Kaplan–Meier method. Survival curves were compared employing the log-rank test.

**Figure 2:**
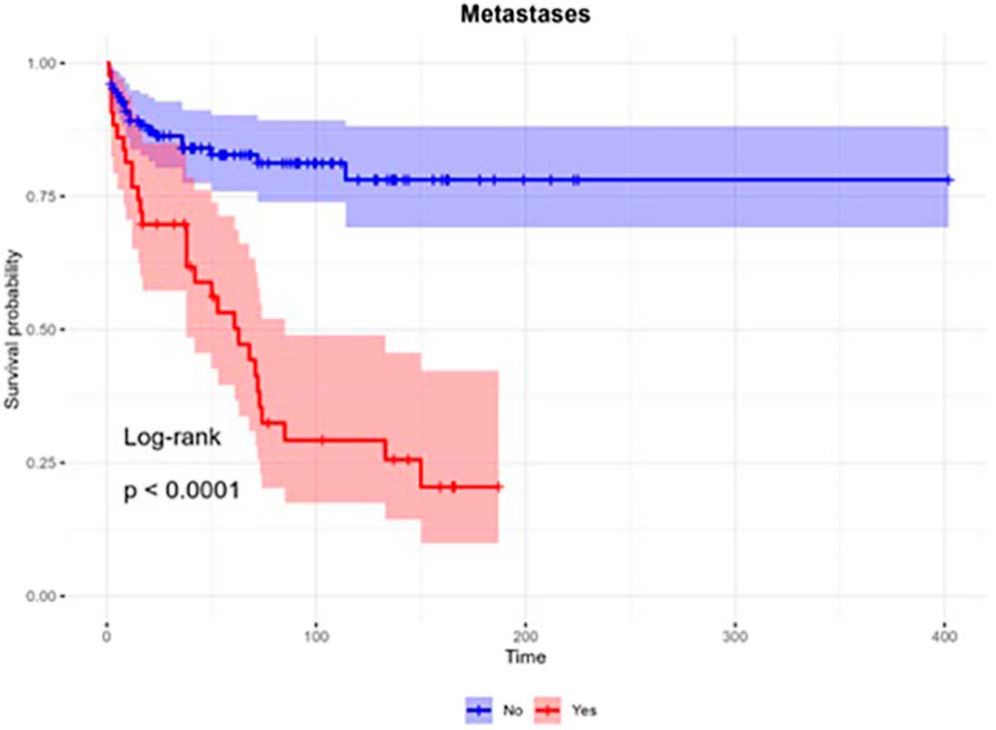
Overall survival by the presence of metastases. Survival probabilities were estimated using the Kaplan–Meier method. Survival curves were compared employing the log-rank test.

**Figure 3:**
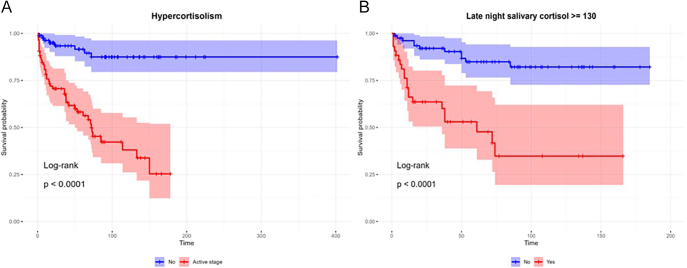
Overall survival by active hypercortisolism and late-night salivary cortisol levels. (A) Hypercortisolism and (B) late-night salivary cortisol. Survival probabilities were estimated employing the Kaplan–Meier method. Survival curves were compared employing the log-rank test.

Statistically significant predictive factors of survival were incorporated into the multivariate Cox regression analysis with a stepwise approach that showed four variables that were significantly (*P*-value  <0.05) associated with mortality among patients with EAS after adjusting for other variables. The final results of the multivariate analysis are presented in Table 4. Mortality among patients aged above 51 at diagnosis was higher than younger patients (Fig. 1). Another important negative predictive factor of survival was the presence of metastasis (Fig. 2). The overall 3- and 5-year survival rates for patients without metastases were 84 and 83% compared with 59 and 41% (*P* < 0.001) in patients with metastases, respectively. Presence of active hypercortisolism and hypercortisolism severity, as indicated by LNSC ≥130 nmol/L, were unfavorable predictors of overall survival (Fig. 3).

**Table 4:** Multivariable Cox regression analysis (stepwise approach) of survival predictors among patients with EAS.

Variable	HR (95% CI)	*P*-value
Presence of metastases	2.93 (1.35–6.32)	0.006
Active hypercortisolism	5.58 (1.62–19.24)	0.006
Age at the time of diagnosis ≥51	3.53 (1.67–7.50)	0.001
Late-night salivary cortisol ≥130 nmol/L	2.81 (1.30–6.07)	0.009

HR, hazard ratio; CI, confidence interval.

## Discussion

We report the outcomes and prognostic factors of survival in 173 Russian patients with EAS, which is, to our knowledge, the largest reported national cohort with the longest observation period. This relatively large-scale study allowed us to perform a multivariate analysis, which revealed that age older than 51 years at the time of diagnosis, presence of metastasis, active hypercortisolism and LNSC above 130 nmol/L are negative predictors of survival.

A previously published multicenter study by Davi *et al.* (16), which included 110 Italian patients with EAS evaluated between 1996 and 2014, showed results similar to our study, such as patients older than 60 years at the time of diagnosis having significantly worse prognosis compared to younger patients. They also reported worse survival rates for patients with metastasis. Davi *et al.* (16) reported that patients with more than a fivefold increase in 24 h UFC had worse survival outcomes in a multivariate Cox model (HR 6.5; 95% CI: 1.5–28.2; *P* = 0.012). In our multivariate analysis, LNSC was a better predictive factor, perhaps because it does not depend on kidney function and urine sample loss, which may be issues in patients with severe hypercortisolism.

In a Finnish cohort of 60 patients observed between 1997 and 2016, negative predictive factors for survival were higher cortisol levels and complications of hypercortisolism, such as hypokalemia, infection and diabetes (3). In our study, the univariate analysis also confirmed that hypercortisolism severity and its complications were negative predictors of survival, whereas bronchial NET localization and successful surgical treatment were positive prognostic factors. However, many prognostic factors closely correlate with each other. For example, severe active hypercortisolism explains hypokalemia and all complications, many of which are reversible (25). We were able to narrow down the multiple predictors of survival related to the severity of hypercortisolism, revealing LNSC with a cut-off value of 130 nmol/L (13.5-fold increase) as the most significant negative predictor for EAS survival.

Several previous studies have described the relationship between the presence of distant metastases and a poor prognosis for patients with EAS (12, 27). In our research, the 5-year survival rate of EAS with the presence of distant metastases was similar to the results of other studies, where the 5-year survival rate ranged from 47 to 60% (12, 16, 27).

According to the literature, bronchial carcinoid is the most common cause of EAS (23, 27, 28), which is supported by the results of the present study. Surgical treatment is the preferred treatment option for EAS (18, 27) and in our study, successful surgical removal of the tumor with established localization lead to remission in 74.4% of cases. Survival rates in patients with occult NETs and pancreatic NETs were comparable in our cohort of patients, in contrast to the results obtained by Ilias *et al.* (10), where patients with occult NETs had better survival rates, almost comparable to those with bronchial carcinoids.

In severe hypercortisolism caused by EAS, bilateral adrenalectomy may be considered if medical control of hypercortisolism fails (13, 29). Almost 30% of patients in our cohort underwent bilateral adrenalectomy, of which 68% performed as the first-line treatment in cases of initially occult tumors. In a previously published study, patients with EAS who underwent adrenalectomy had a better survival rate in the first 2 years, highlighting the importance of rapid control of hypercortisolism (16). We did not find any difference in survival rates between those who underwent and those who did not undergo bilateral adrenalectomy. However, as the presence of hypercortisolism is a negative predictor of survival, we may assume that urgent hypercortisolism resolution through different approaches (primary tumor removal, medical control or bilateral adrenalectomy) may be beneficial.

Although this study evaluates, to our knowledge, the largest national cohort of patients with EAS over 34 years of observation, we acknowledge the retrospective design of the study, with all the limitations associated with these designs, including data omission, changes in patient management over time, and changes in tumor classifications, which may have influenced outcomes. In 26 cases of occult tumors, diagnosis was based on BIPSS with desmopressin stimulation. Nevertheless, BIPSS is currently reported as the most accurate test to differentiate EAS from CD (30). In our cohort, bilateral adrenalectomy was performed in the most severe life-threatening cases, which is another limitation of the present study. In addition to this, LNSC as the main biochemical predictor may be influenced by variability of available assay techniques. The provided threshold is only meaningful for the ECLIA method performed on the Roche manufacturer (17).

In conclusion, the negative factors associated with higher mortality in EAS are active hypercortisolism, high LNSC, presence of metastases and older age at diagnosis. As the severity of hypercortisolism is the main targetable factor, this should be the focus of intervention and further studies to improve patient outcomes.

## Supplementary materials



## Declaration of interest

The authors declare that the work was conducted in the absence of any commercial or financial relationships that could be construed as a potential conflict of interest.

## Funding

This work was supported by the Russian Science Foundationhttps://doi.org/10.13039/501100006769 (RSF Grant No. 24-15-00283).
