# Supplementary material for: Long-term outcomes and survival predictors in patients with ectopic ACTH syndrome: data from a retrospective cohort study
Source: Endocr Connect. 2025 Sep 22;14(9):e250411. doi: 10.1530/EC-25-0411 (PMC12464338; doi:10.1530/EC-25-0411)
Supplement: Supplementary file 1 [file supplementary_materials.pdf]

Supplementary Table 1.
Comparative analysis of younger (18-35) and older (36+) adults.

Parameter		Group 1<br>N = 57	Group 2<br>N = 111	p-value
Male / Female		30 / 27	34 / 77	0.007
Age at the time of diagnosis, years		Me 27 [23;30]	Me 53 [42;60]	< 0.001
Body mass index		Me 26 [22.9;30.3]	Me 28.8 [25.9;33.2]	0.001
Survived / Died		45 / 11	68 / 39	0.032
NET localization	Bronchial carcinoid	36 (63.2%)	69 (62.2%)	NS
	Thymic carcinoid	9 (15.8%)	7 (6.3%)	NS
	Pheochromocytoma	1 (1.8%)	5 (4.5%)	NS
	Pancreatic NET	3 (5.3%)	6 (5.4%)	NS
	Cecum NET	–	1 (0.9%)	NS
	Appendix NET	1 (1.8%)	–	NS
	Medullary thyroid carcinoma	–	1 (0.9%)	NS
	Renal NET	–	3 (2.7%)	NS
	Occult cases	7 (12.3%)	19 (17.1%)	NS
Ki-67 index, %		3.9 [1.9;13.9]	3.0 [1.5;7.8]	0.112
Metastases	Yes	14 (24.6%)	29 (26.1%)	0.854
	No	43 (75.4%)	82 (73.9%)	
**Complications in the active stage of disease**				
Arterial hypertension	Yes	38 (66.7%)	107 (96.4%)	< 0.001
	No	19 (33.3%)	4 (3.6%)	
Cardiovascular disease	Yes	20 (35.1%)	72 (64.9%)	< 0.001
	No	37 (64.9%)	39 (35.1%)	
Diabetes mellitus	Yes	24 (42.1%)	74 (66.7%)	0.003
	No	33 (57.9%)	37 (33.3%)	
Osteoporosis	No osteoporosis	28 (49.1%)	42 (37.8%)	0.311
	Osteoporosis without low-energy fractures	21 (36.8%)	54 (48.6%)	
	Osteoporosis with low-energy fractures	8 (14.0%)	15 (13.5%)	
**Laboratory examinations at the time of diagnosis**				
Morning ACTH, pg/mL		156.4 [106.2;213.1]	136 [102.6;203.1]	0.144
Late-night ACTH, pg/mL		134.9 [101.4;190.7]	114.7 [78.1;177.5]	0.117
Late-night salivary cortisol, nmol/L		77.4 [45.9;124.8]	90.1 [46.5;202.9]	0.201
24-h urinary free cortisol, nmol/24h		3694.5 [1970;7000]	2817.5 [1736.3;5694.4]	0.191
Late-night serum cortisol, nmol/L		1130 [832.3;1461.3]	1160.5 [986.5;1389]	0.578

Group 1:18–35 years old (total 57 patients, data on survival available in 56 cases, 1 case lost to follow-up)
Group 2: 36–76 years old (total 111 patients, data on survival available in 107, 4 cases lost to follow-up)
